# Supplementary material for: From sequence to enzyme mechanism using multi-label machine learning
Source: BMC Bioinformatics. 2014 May 19;15:150. doi: 10.1186/1471-2105-15-150 (PMC4229970; doi:10.1186/1471-2105-15-150)
Supplement: Additional file 2 — Java code of ml2db. Additional file ml2db_code.tar.gz contains the Java source code to run the multi-label machine learning experiments and save the results to database. The code’s Javadoc is included. [file 1471-2105-15-150-S2.zip › additional file 2/ml2db/ml2db/doc/main/ml2db/al/database/AlDbCreator.html]

AlDbCreator


JavaScript is disabled on your browser.


- Overview
- Package
- Class
- Use
- Tree
- Deprecated
- Index
- Help

- Prev Class
- Next Class

- Frames
- No Frames

- All Classes

- Summary:
- Nested |
- Field |
- Constr |
- Method

- Detail:
- Field |
- Constr |
- Method


main.ml2db.al.database

## Class AlDbCreator

- java.lang.Object
- - uk.ac.standrews.utils.main.database.DbManaged
  - - uk.ac.standrews.utils.main.database.DbCreator
    - - main.ml2db.al.database.AlDbCreator

- ---

    

  ```
  public class AlDbCreator
  extends uk.ac.standrews.utils.main.database.DbCreator
  ```

  Creates the tables to store Active Learning machine learning experiments and
  results. For the tables to be created, an empty database (with the name
  specified in the datbase connection properties file) must already have been
  created and a user with reading/writing rights must be available.
  Tables: (1) experiments (experiment id, distance function, utility function,
  unlabelled and labelled arffs etc.), (2) experiments steps (utility metric
  for each instance at each active learning step), (3) query instances (the
  instance picked at each step to be labelled) and (4) the evaluation metrics
  when training on the labelled set and testing on the unlabelled set.

  Version:
  :   12 Jul 2011

  Author:
  :   Luna De Ferrari ldf(at)st-andrews.ac.uk

- - ### Field Summary

    Fields

    | Modifier and Type | Field and Description |
    | --- | --- |
    | `static java.lang.String` | `EXPERIMENT_ID` the table column to store the unique experiment identifier |
    | `static java.lang.String` | `INSTANCE` the instance unique name |
    | `static java.lang.String` | `STEP_ID` the table column to store the step identifier (one step is what takes to obtain one query instance) |
  - ### Constructor Summary

    Constructors

    Constructor and Description

    `AlDbCreator(uk.ac.standrews.utils.main.database.DbManager dbManager)`
  - ### Method Summary

    Methods

    | Modifier and Type | Method and Description |
    | --- | --- |
    | `void` | `createTables()` |

    - ### Methods inherited from class uk.ac.standrews.utils.main.database.DbCreator

      `dropTable, getSqlToDropTable`
    - ### Methods inherited from class uk.ac.standrews.utils.main.database.DbManaged

      `getDbConnection, getDbManager, getDbReader`
    - ### Methods inherited from class java.lang.Object

      `equals, getClass, hashCode, notify, notifyAll, toString, wait, wait, wait`

- - ### Field Detail


    - #### STEP\_ID

      ```
      public static final java.lang.String STEP_ID
      ```

      the table column to store the step identifier (one step is what takes to
      obtain one query instance)

      See Also:
      :   Constant Field Values


    - #### INSTANCE

      ```
      public static final java.lang.String INSTANCE
      ```

      the instance unique name

      See Also:
      :   Constant Field Values


    - #### EXPERIMENT\_ID

      ```
      public static final java.lang.String EXPERIMENT_ID
      ```

      the table column to store the unique experiment identifier

      See Also:
      :   Constant Field Values
  - ### Constructor Detail


    - #### AlDbCreator

      ```
      public AlDbCreator(uk.ac.standrews.utils.main.database.DbManager dbManager)
      ```
  - ### Method Detail


    - #### createTables

      ```
      public void createTables()
      ```

      **Overrides:**
      :   `createTables` in class `uk.ac.standrews.utils.main.database.DbCreator`


- Overview
- Package
- Class
- Use
- Tree
- Deprecated
- Index
- Help

- Prev Class
- Next Class

- Frames
- No Frames

- All Classes

- Summary:
- Nested |
- Field |
- Constr |
- Method

- Detail:
- Field |
- Constr |
- Method
